# Impact of Intestinal Microbiota on Cognitive Flexibility by a Novel Touch Screen Operant System Task in Mice

**DOI:** 10.3389/fnins.2022.882339

**Published:** 2022-06-23

**Authors:** Hazuki Tamada, Kayo Ikuta, Yusuke Makino, Daisuke Joho, Takeru Suzuki, Masaki Kakeyama, Mitsuharu Matsumoto

**Affiliations:** ^1^Dairy Science and Technology Institute, Kyodo Milk Industry Co., Ltd., Tokyo, Japan; ^2^Laboratory for Environmental Brain Sciences, Faculty of Human Sciences, Waseda University, Tokorozawa, Japan; ^3^Research Institute for Environmental Medical Sciences, Waseda University, Tokorozawa, Japan

**Keywords:** touch screen operant system, serial reversal learning, behavioral sequencing task, streptomycin, Firmicutes, Bacteroidetes

## Abstract

Cognitive flexibility is the ability to rapidly adapt to a constantly changing environment. It is impaired by aging as well as in various neurological diseases, including dementia and mild cognitive impairment. In rodents, although many behavioral test protocols have been reported to assess learning and memory dysfunction, few protocols address cognitive flexibility. In this study, we developed a novel cognitive flexibility test protocol using touch screen operant system. This test comprises a behavioral sequencing task, in which mice are required to discriminate between the “rewarded” and “never-rewarded” spots and shuttle between the two distantly positioned rewarded spots, and serial reversals, in which the diagonal spatial patterns of rewarded and never-rewarded spots were reversely changed repetitively. Using this test protocol, we demonstrated that dysbiosis treated using streptomycin induces a decline in cognitive flexibility, including perseveration and persistence. The relative abundances of Firmicutes and Bacteroides were lower and higher, respectively, in the streptomycin-treated mice with less cognitive flexibility than in the control mice. This is the first report to directly show that intestinal microbiota affects cognitive flexibility.

## Introduction

Cognitive flexibility is a core component of executive function, inhibition, and working memory (Diamond, 2013) and the behavioral ability to rapidly adapt to a constantly changing environment. Reportedly, cognitive flexibility is impaired by aging and dementia, especially in Alzheimer’s disease (AD), frontal lobe dementia and mild cognitive impairment (Albert, 1996; Wecker et al., 2005; Tatsuoka et al., 2013; Berry et al., 2016; Guarino et al., 2019).

Tools, such as the Wisconsin card sorting test (Nelson, 1976; Nagahama et al., 2003; Nordlund et al., 2007), the Cambridge neuropsychological test automated battery (CANTAB) (Blackwell et al., 2004; Egerhazi et al., 2007), and the Brixton spatial anticipation test (BSAT) (Burgess and Shallice, 1997; Caso and Cooper, 2022) have been developed to evaluate cognitive flexibility in humans. These tests measure the ability to recognize changes in existing rules and adapt to new rules, allowing evaluation that correlates with mild cognitive impairment and dementia especially in severity of AD (Fowler et al., 1997; Swainson et al., 2001; Nagahama et al., 2003; Egerhazi et al., 2007; Kessels et al., 2009; Kuzmickiene and Kaubrys, 2016).

Cognitive flexibility in mice has been assessed by using reversal tasks based on spatial or visual discrimination (Brigman et al., 2008, 2009; Karlsson et al., 2009; Horner et al., 2013; Oomen et al., 2013; Romberg et al., 2013; Yang et al., 2015). It is suggested that mice acquire a reversal rule only as a novel rule and are unable to develop a reversal learning-set, as mice showed less or no ability to improve progressively in serial reversals (Endo et al., 2011).

We previously established the test protocol to assess cognitive flexibility in mice using IntelliCage, a fully automated behavioral analysis system for a group-housed mice (Endo et al., 2011). The flexibility test protocol in IntelliCage is composed of a behavioral sequencing task followed by serial reversal tasks. A mouse first acquires behavioral sequencing, wherein the mouse must distinguish between two “rewarded” and two “never-rewarded” locations and shuttle between the two distantly positioned diagonal rewarded locations to obtain drinking water as a reward. In serial reversal tasks based on its behavioral sequencing, the diagonal spatial positions of the rewarded and never-rewarded corners are reversed. In this task, mice succeeded to show progressive improvement over a series of reversals (Endo et al., 2011, 2012). This rapid adaptation to reversal learning observed in IntelliCage indicates that mice can show a long term learning effect which was attributed to an adaptation to “reversal rule” itself, or a reversal “learning-set.”

Recent studies have revealed that gut microbiota impact the gut-brain axis, which influences the bidirectional signaling between the gastrointestinal tract and the brain *via* the nervous system, neurotransmitters, and so on, a concept termed the “microbiota-gut-brain axis” (Rhee et al., 2009; Collins et al., 2012; Cryan and Dinan, 2012; Sharon et al., 2016). Germ-free mice exhibit a different brain metabolome to that of normal mice (Matsumoto et al., 2013) and exhibit decreased anxiety and social behaviors (Diaz Heijtz et al., 2011; Sharon et al., 2016). Additionally, rodents with dysbiosis treated by antibiotics also change their behaviors. Specifically, increase in exploratory behavior, induction of cognitive deficits, and impairments in novel object recognition as well as anxiety-like and social behaviors were reported (Bercik et al., 2011; Desbonnet et al., 2015; Frohlich et al., 2016; Leclercq et al., 2017). From these results, it is generally understood that there is a strong relationship between the gut microbiome and symptoms related to neurodevelopmental disorders, such as anxiety-like behavior, depression, and autism. However, there is controversy regarding the influence of the gut microbiome on cognitive function, for instance, in Frohlich’s report, the gut microbiome is reported to influence novel object recognition, but not cognitive function (Frohlich et al., 2016). To the best of our knowledge, there has been no study demonstrating the apparent relationship between gut microbiome and cognitive flexibility. One study describes the relationship between cognitive flexibility and gut microbiota using mice with gut microbiota that has been altered by different diets (Magnusson et al., 2015). However, a possibility remains that factors other than gut microbiota, such as the obesity caused by diets with high fat or sucrose, contribute to this phenomenon.

In the present study, we developed a new task for cognitive flexibility with a touch screen operant system based on IntelliCage (NewBehavior AG, Zurich, Switzerland) as a high-throughput system that is used for behavioral screening and detailed analyses of complex behaviors in mice (Endo et al., 2011). We then attempted to analyze the influence of the gut microbiome on cognitive flexibility using this system. We hypothesized that dysbiosis would reduce cognitive flexibility *via* the microbiota-gut-brain axis and analyzed the influence of the gut microbiome on cognitive flexibility using this system.

## Materials and Methods

### Animals

The animal experiment was conducted in accordance with protocols approved by the Kyodo Milk Industry Animal Use Committee (permits 2017-011). Male C57BL6/J mice (8 weeks old) were purchased from Charles River Laboratories Japan, Inc. (Yokohama, Japan). All mice were housed in a temperature- and light-controlled environment (25 ± 1°C, 50% humidity, 12-h light/dark cycle) under conventional conditions. All mice had free access to CRF-1 chow (Oriental Yeast Co., Tokyo, Japan) until the commencement of dietary restrictions.

### Antibiotic Treatment for Gut Microbiota Alteration

At 12 weeks of age, the male C57BL6/J mice were randomly divided into two groups (*n* = 5 per group): control mice and streptomycin-treated mice. The latter were treated with 2.0 mg/mL streptomycin in drinking water from 2 weeks prior to the behavioral test until the end of the test. The drinking water was changed twice weekly. Streptomycin is an aminoglycoside antibiotic that has antibacterial activity against both Gram-negative and Gram-positive bacteria, and was selected for our experiment based on the fact that this is poorly absorbed when given orally, minimizing the risk of systemic effects on the host (Germovsek et al., 2017). The antibiotic treatment was continued during the examination period. All mice had free access to drinking water.

### Touch Screen Operant System Apparatus

**FIGURE 1:**
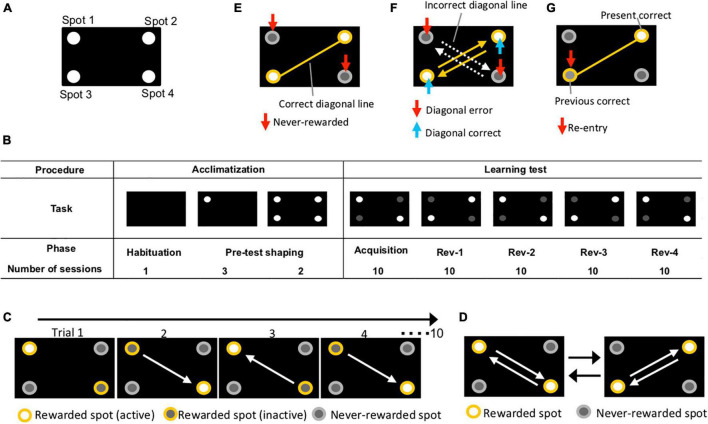
Overview of experiment using touch screen operant system. **(A)** Position of spots. Four holes were cut in an acryl plate and the plate was placed on a touch screen. **(B)** Experimental procedure. Acclimatization comprised “habituation” and “pre-test shaping” (1 and 5 days, respectively). The learning test included “acquisition” and “reversal” stages 1, 2, 3, and 4 (10 sessions/phase). **(C)** Diagrams of behavioral sequencing task. Mice obtained a reward by alternately touching the two distantly positioned reward spots (yellow open circles). **(D)** Serial reversal learning of the behavioral sequencing task. The pattern of corner conditions (rewarded or never-rewarded) was reversely switched for each phase. The white arrows indicate the expected shuttling path on which mice shuttle between positioned rewarded spots. **(E)** Never-rewarded. A nose-poking of the gray circles, which is not on the correct diagonal line, was counted as “never-rewarded” choice (red arrows). Results of first-choice never-rewarded rates and never-rewarded rates of the first 100 trials are shown in Figures 3, 4B, respectively. **(F)** Diagonal correct; diagonal error. Shuttle nose-poking (blue arrows) of rewarded spots (yellow spots) on the correct diagonal line (yellow lines) was counted as “diagonal correct” choice. Shuttle nose-poking (red arrows) of never-rewarded spots (gray spots) on the incorrect diagonal line (white dot arrows) was counted as “diagonal error” choice. Data of cumulative diagonal correct and error are shown in Figure 4A. **(G)** Re-entry. Nose-poking of the incorrect spot (red arrows) on correct diagonal line (yellow line), the last correct spot, was counted as “re-entry.” Results of re-entry rate are shown in Figures 2, 3D.

Experiments were conducted in a touch screen operant chamber for mouse (O’Hara & Co., Ltd., Tokyo, Japan). The apparatus was regulated by computer and was composed of a monitor, touch screen, pellet dispenser, a water bottle, a speaker, a house lamp, a camera, and a chamber. The mice could receive a reward pellet when they reacted directly to the cue on the screen. The position of spots is shown in Figure 1A.

**FIGURE 2:**
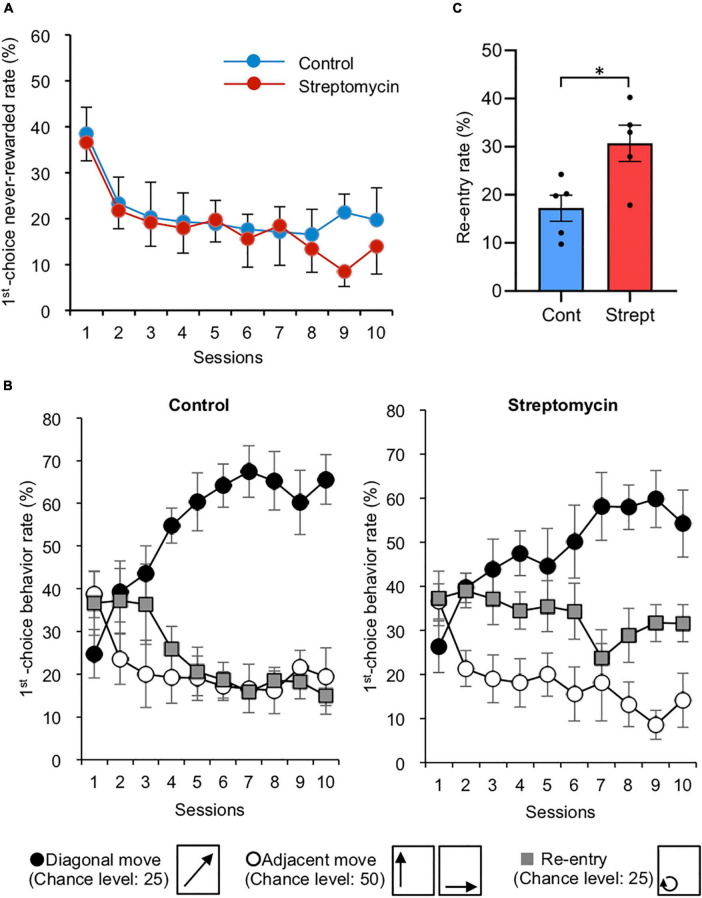
Effects of streptomycin treatment during acquisition phase (sessions 1–10). **(A)** Change in first-choice never-rewarded rate (*n* = 5/group). Error bars represent standard error of the mean (SEM). This rate was defined as the number of nose-pokes to the two never-rewarded points (described in Figure 1E) within the first 100 trials in each session, which makes the chance value of this error rate 50%. **(B)** The rates of the three move patterns in nose-poking of two consecutive points during the acquisition phase (*n* = 5/group). These movements were classified as follows: (1) diagonal move, (2) adjacent move (move to either of two adjacent points), and (3) re-entry to the same corner. Thus, chance values are calculated as 25, 50, and 25%, respectively (these movements are shown below the graph). Error bars represent SEM. **(C)** Re-entry rate (%) of average of final three sessions of acquisition phase. ^∗^*p* < 0.05 by Student’s *t*-test.

**FIGURE 3:**
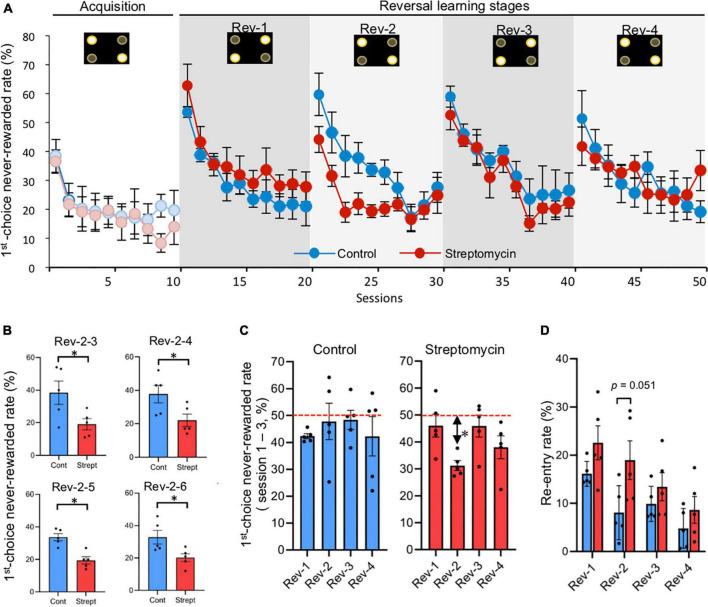
Differences of first-choice never-rewarded rate between control mice and streptomycin-treated mice during serial reversal learning stage (sessions 11–50). **(A)** Change of first-choice never-rewarded rate (described in Figure 1E) of both control mice and streptomycin-treated mice are shown (*n* = 5/group). Error bars represent SEM. Active and inactive spots (described in Figure 1C) are shown in the upper part of graph in each stage. **(B)** The difference between groups of the first choice never-rewarded rates in each session in the reversal stage. Only four sessions (Rev-2-3, 4, 5, and 6) showed significant differences. ^∗^*p* < 0.05 by two-way repeated measures ANOVA with Bonferroni correction. **(C)** Comparison of first-choice never-rewarded rate in first three sessions of each reversal stage (data of Rev-1, Rev-2, Rev-3, and Rev-4 are the averages of Rev-1-1 to 3, Rev-2-1 to 3, Rev-3-1 to 3, and Rev-4-1 to 3, respectively, in each group). ^∗^*p* < 0.05, significant difference from chance level (50%) using Student’s one-sample *t*-test. Error bars represent SEM. Horizon dotted lines represent 50% chance level. **(D)** Re-entry rate (described in Figure 1G) in first three sessions of each reversal stage (data of Rev-1, Rev-2, Rev-3, and Rev-4 are the averages of Rev-1-1 to 3, Rev-2-1 to 3, Rev-3-1 to 3, and Rev-4-1 to 3, respectively, in each group). *P* value between-group comparison was obtained by two-way repeated measures ANOVA with Bonferroni correction.

**FIGURE 4:**
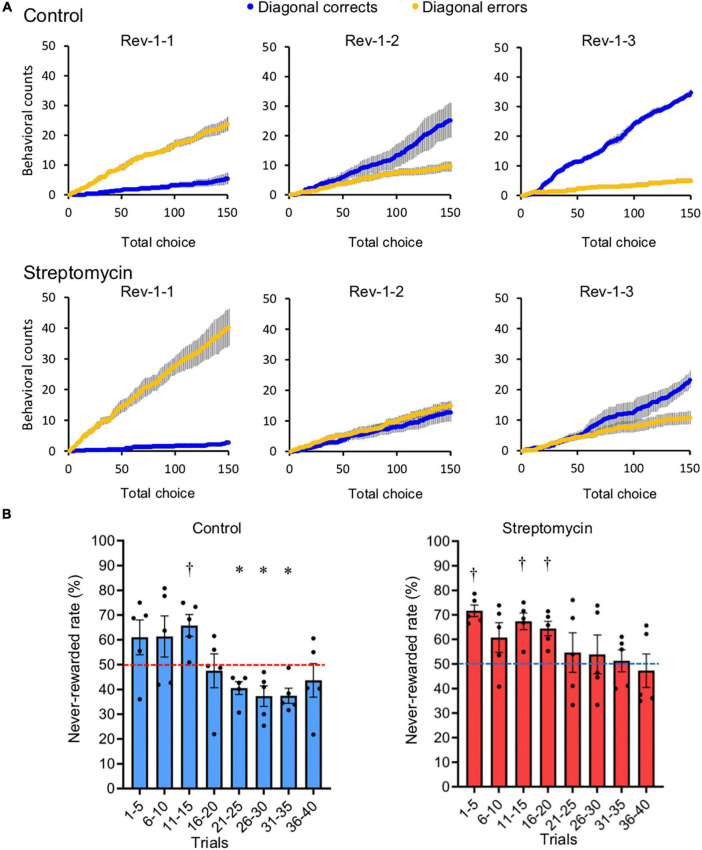
Comparison of adaptation speed to the reversed contingency. **(A)** Cumulative diagonal correct (blue) and error (yellow) counts (described in Figure 1F) within the first 150 choices in each of the first three sessions of Rev-1 (Rev-1-1 to Rev-1-3) (*n* = 5/group). **(B)** Never-rewarded rates in every 5-trial block of the first 40 trials, which all mice completed, in the first session of Rev-1 (Rev-1-1). ^∗^,^†^*p* < 0.05, significantly lower (^∗^) or higher (^†^) from chance level (50%) using Student’s one-sample *t*-test. Horizontal dotted lines represent 50% chance level. Error bar represents SEM of never-rewarded rates compiled for every five-trial block. This value did not significantly decrease below chance level in streptomycin-treated mice.

### Cognitive Flexibility Test Protocols

We used the learning test protocol described in Figure 1B. All mice were bred on CRF-1 and were fasted for 12 h before starting the acclimatization. During the test period, the diet was restricted to 2.2 g/day, including the reward pellets (AIN-76A Rodent Tablet #1811213, TestDiet, St. Louis, MO, United States) and normal pellets (CRF-1). The dietary restriction was continued during the examination period to keep their body weight at approximately 80% compared to non-tested mice, which were of the same age and sex and had *ad libitum* feeding, and thereby maintained their motivation for rewards. Through the preliminary test, we confirmed that 2.2 g of intake maintained body weight and motivation for rewards. Body weight was measured daily after the end of the behavioral test. All sessions were performed once a day.

### Acclimatization

Acclimatization consisted of a phase of habituation and pre-test shaping, which lasted for 1 and 5 days, respectively (Figure 1B). Mice were habituated to the apparatus and rewarded with pellets during the habituation phase. Two habituation conditions were implemented as follows: Habituation 1: mice could explore the internal chamber and intake five reward pellets for 15 min; Habituation 2: the apparatus delivered one reward pellet every 30 s for 15 min with sound when dispensing the reward pellet. The habituation phase lasted for one session of 30 min, which was composed of habituation conditions 1 and 2. The following day, the pre-test shaping phase was started, wherein mice learned that to receive a reward pellet they must poke the screen. In pre-test shaping condition 1, mice received a reward pellet with sound if they nose-poked lighting spot 1 on the screen (Figure 1B). This action was defined as one trial, and one session was composed of 100 trials. The session lasted 45 min, but if the mice could not finish 100 trials within the time limit, the session was aborted at 45 min. Pre-test shaping condition 1 was performed once a day for 3 days. In pre-test shaping condition 2, mice received a reward pellet with sound if they nose-poked lighting spot 1, 2, 3, or 4 on the screen (Figure 1B). This action was defined as one trial, and one session was composed of 100 trials. The session lasted for 45 min, but if mice could not finish 100 trials within the time limit, the session was aborted at 45 min. Pre-test shaping condition 2 was performed once a day for 2 days.

### Behavioral Sequencing Task

After acclimatization, the behavioral sequencing task was conducted. In the behavioral sequencing task, mice were required to discriminate between the “rewarded” and “never-rewarded” spots and to shuttle between the two distantly positioned rewarding spots (Figure 1C). Each mouse received a reward pellet only when the mouse nose-poked the spots designated “rewarded,” while they could not do so in the “never-rewarded” spots. Additionally, each of the two distantly positioned rewarded spots had two distinct states, “active” or “inactive,” in a mutually exclusive manner. That is, there was always one active rewarded spot and one inactive rewarded spot, and two never-rewarded spots, at a time. The reward pellet could be provided by a nose-poke action only in an active rewarded spot, then the spot became inactive. At the same time, the other rewarded spot, which was previously inactive, became active. All the spot assignments were counterbalanced within a group so that no specific spot had concentrated preference. As an “acquisition” stage, this behavioral sequencing task lasted for 10 sessions (Figure 1B). One session was composed of 100 trials and the session lasted for 45 min, but if mice could not finish 100 trials within the time limit, the session was aborted at 45 min. One trial lasted until the mouse selected the active rewarded spot out of the four spots.

### Serial Reversal Learning

A serial reversal task using the behavioral sequencing task paradigm was conducted: diagonal spatial patterns of rewarded and never-rewarded spots were reversely changed repetitively every 10 sessions (Figure 1D). Thus, the mice had to learn to switch their shuttling behavior between the two diagonal spatial patterns.

### Behaviors to Score

The records during each session were used to calculate the following five learning scores: (1) The rates of three move patterns in nose-poking of two consecutive spots during the acquisition phase: diagonal move (the first choice in a trial, move diagonally from the correct spot in the immediately preceding trial to the following correct spot), adjacent move (the first choice in a trial, move to either of the two spots adjacent to the correct spot in the immediately preceding trial), and re-entry (the first choice in a trial, nose-poke to the same spot as the previous correct spot in the immediately preceding trial) (Figure 2B). (2) First-choice never-rewarded rate: percentage of nose-poking to never-rewarded spots, which are not on the correct diagonal line, at first choice of the trial (Figure 1E). (3) Cumulative diagonal correct and error count: alternate nose-poking of rewarded spots on the correct diagonal line was counted as a “diagonal correct” choice. Alternate nose-poking of never-rewarded spots on the incorrect diagonal line was counted as a “diagonal error” choice. Each choice was counted cumulatively (Figure 1F). (4) Never-rewarded rate: percentage of nose-poking to never-rewarded spots, which are not on the correct diagonal line, out of all nose-poking. In this study, this value was obtained from every 5-trial blocks of 100 trials in the first session of Rev-1 (Rev-1-1). (5) First-choice re-entry rate (re-entry): percentage of nose-poking to the same spot as the previous correct spot in the immediately preceding trial, at first choice of the trial (Figure 1G). Scoring system of the behaviors is shown in Supplementary Table 2.

### Extraction of Bacterial DNA From Feces

The feces of mice were collected after finishing all the behavioral tests and stored at −80°C. Bacterial DNA was extracted using a modified version of the methods described by Matsuki et al. (2004). Frozen fecal samples (approximately 50 mg) were diluted 10-fold with Dulbecco’s phosphate buffered saline (pH 7.2) (D-PBS, Thermo Fisher Scientific, Waltham, MA, United States) and suspended three times with intense mixing for 1 min using a vortex mixer followed by incubation on ice for 5 min. The upper aqueous portion (200 μl) was collected to exclude large precipitates and centrifuged at 13,000 × *g* for 5 min at 4°C to collect the bacterial precipitates. Bacterial precipitates were diluted in nuclease-free water (200 μl), followed by the addition of 0.1 mm beads (300 mg), bacterial lysis buffer (100-mM Tris–HCl/50-mM EDTA, pH 8, 350 μl), 10% sodium dodecyl sulfate (50 μl), and phenol (500 μl). Protein was denatured for 10 min at 70°C and then homogenized for 1 min at 4,000 rpm followed by centrifugation at 15,000 × *g* for 5 min. The supernatant (500 μl) was mixed with phenol:chloroform:isoamyl alcohol (25:24:1, 350 μl) and centrifuged at 15,000 × *g* for 5 min. The supernatant, which included the DNA, was purified using Ethachinmate (Nippongene, Toyama, Japan), subsequently air-dried at room temperature overnight, and diluted with Milli-Q water (500 μl). Bacterial DNA solutions were diluted 10-fold and then used for 16S rRNA gene analysis.

### Analysis of Intestinal Microbiota

The V1–V2 regions of bacterial 16S rRNA genes were amplified using polymerase chain reaction (PCR) with fusion primers. The forward primer contained an ion A adapter sequence followed by key, barcode, adapter (GT) and 27Fmod primer sequences (Kim et al., 2013). The reverse primer contained an ion-truncated P1 adapter sequence, followed by adapter (CC) and 338R primer sequences. PCR was performed in 25 μl reaction mixtures containing 23.5 μl Platinum^®^ PCR SuperMix High Fidelity (Invitrogen), 0.5 μl primer mixture (5 μM each primer) and 1 μl DNA solution. Thermal cycling conditions were 3 min at 94°C, followed by 25 cycles of 30 s at 94°C, 45 s at 55°C, and 1 min at 68°C. The amplicon was purified using a PureLink PCR Purification Kit (Invitrogen). Purified samples were then loaded onto 2.0% agarose gels and electrophoresed at 50 V for 1.5 h at 25°C. Target DNA fragments were excised from the gel and purified using a FastGene Gel/PCR Extraction Kit (Nippon Genetics, Tokyo, Japan). The purity and concentration of DNA samples were assessed using a bioanalyzer (Agilent Technologies, Santa Clara, CA, United States) and a high-sensitivity DNA kit (Agilent). Molecular concentrations were adjusted according to the Ion Torrent protocol (Life Technologies, Gaithersburg, MD, United States). Emulsion PCR was performed using an Ion PGM Template OT2 400 Kit (Life Technologies) according to the manufacturer’s instructions. Library sequencing was performed using the Ion PGM Sequencing 400 Kit (Life Technologies) and Ion 318 Chip V2 (Life Technologies) according to the manufacturer’s instructions. Sequencing was performed on an Ion PGM System (Life Technologies).

Sequence data were obtained in fastq format and analyzed using QIIME software (Caporaso et al., 2010b). Raw sequences were sorted according to barcode and then screened for quality (average quality score ≥20) and primer sequence correctness. The trimmed sequences were clustered into operational taxonomic units (OTUs) at a level of 97% similarity using the UCLUST method (Edgar, 2010) and the farthest neighbor algorithm. The most abundant sequences in each OTU were selected as representatives. Representative sequences were aligned with the PyNAST algorithm (Caporaso et al., 2010a) and verified for potentially chimeric sequences using the ChimeraSlayer algorithm. Non-chimeric sequences were assigned taxonomy using Ribosomal Database Project classifiers with a confidence cut-off value of 80 (Wang et al., 2007).

### Statistical Analysis

All statistical analyses for difference in behavioral score were performed using SPSS Statistics version 22 (IBM, North Castle, NY, United States). The behavioral data and body weight data were compared using two-way repeated measures ANOVA, using the Bonferroni correction, Student’s *t*-test, or Student’s one-sample *t*-test. Principle component analysis (PCA) of fecal microbiota (relative abundance of each taxonomic rank) data was performed using SPSS Statistics version 22. The relative abundance ratio of fecal bacteria was compared using Mann–Whitney *U*-test and corrected using the Benjamini–Hochberg method (Benjamini and Hochberg, 1995) using R statistical software version 3.4.4 (R Foundation for Statistical Computing, Vienna, Austria). Error bars indicate standard errors. *P* values < 0.05 were considered to be statistically significant.

## Results

### Body Weight and Feeding

A significant difference in body weight between the two groups was not found, although the body weights of streptomycin-treated mice were slightly lower than that of control mice throughout the entire test period (Supplementary Figure 1). Temporary weight loss was observed without any statistical significance in both groups at the start of the task, but was recovered within a few days. In addition, although the amount of feed given was limited because it was a test based on food as reward, body weight did not decrease, and an increase of 1–3 g was observed in all individuals during the test period. During the test period, some mice left a portion of the reward pellets in the touch screen operant system, despite completing 100 trials. For those mice, the amount of normal pellets provided after their testing was reduced to keep them motivated for the reward pellets for future trials. As a result, the mice no longer left any reward pellets and maintained their body weight at approximately 80% of the weight of non-tested mice.

### Behavior Profile of Touch Screen Operant Task

#### Acquisition of Behavioral Sequencing Task

In the behavioral sequencing task, mice were required to discriminate “rewarded” spots from “never-rewarded” spots with attainment of shuttling behavior between the two diagonally positioned rewarded spots to obtain pellets continuously (Figure 1C). First nose-poking to the two never-rewarded spots in each trial was defined as a “first-choice never-rewarded.” The rate of first-choice never-rewarded, which is equal to the number of first-choice never-rewarded within the first 100 trials in each session, was decreased in both groups (36.6 ± 4.0% at the first session to 14.0 ± 6.0% at the final session in the streptomycin-treated mice; 38.5 ± 5.7% at the first session to 19.7 ± 6.9% at the final session in the control mice, chance level is calculated as 50%) (Figure 2A).

To analyze the attainment of shuttling behavior, three move patterns in two consecutive spot visits were classified as follows: (1) diagonal move, (2) adjacent move (move to either of two adjacent spots), and (3) re-entry to the same spot. Thus, chance levels were calculated as 25, 50, and 25%, respectively. The rates of the three move patterns in the acquisition phase of both groups are representatively shown in Figure 2B. The diagonal move rate increased in both the control group (24.8 ± 5.6 to 65.6 ± 5.8%) and the streptomycin-treated group (26.3 ± 5.9 to 54.2 ± 7.6%). Adjacent move rate decreased in both the control group (38.7 ± 5.5 to 19.4 ± 6.8%) and the streptomycin-treated group (36.5 ± 4.0 to 14.1 ± 6.1%). Although the re-entry rate of control mice tended to decrease (36.6 ± 7.4 to 15.0 ± 4.3%), no change was found in the streptomycin-treated mice (37.2 ± 6.2 to 31.6 ± 4.3%) and this value of the average of final three sessions was significantly higher in streptomycin-treated mice than in the control ones (*p* = 0.02, Student’s *t*-test, Figure 2C).

#### Serial Reversal Learning

In serial reversal learning using the behavioral sequencing task paradigm, diagonal patterns of rewarded and never-rewarded spots were reversely changed every 10 sessions. Thus, the mice were expected to switch their shuttling behavioral sequence between the two diagonals (Figure 1D). We analyzed the first-choice never-rewarded rates for each session to clarify the inter-session dynamics of cognitive flexibility (Figure 3A and Supplementary Table 3). In comparison with the last session (session 10) of the acquisition phase and the first session of the first reversal stage (Rev-1-1; session 11), the first-choice never-rewarded rates in control mice (53.6 ± 1.8%) and streptomycin-treated mice (62.8 ± 7.4%) were found to be markedly increased to more than 50%, exceeding the chance level (=50%). Subsequently, the number of sessions that were required for these rates to drop to below 30% in streptomycin-treated mice (Rev-1-8; session 18) tended to be longer than that in the control mice (Rev-1-4; session 14). In the second reversal stage (Rev-2), the first-choice never-rewarded rates of the first session (Rev-2-1; session 21) in control mice were increased to 59.7 ± 7.3% compared to the final session of the Rev-1 (Rev-1-10; session 20). In contrast, these rates in streptomycin-treated mice were only increased to 44.1 ± 4.4%, which is lower than chance level. The number of sessions that were required to drop to below 30% in streptomycin-treated mice was shorter than that in control mice. In addition, these values were significantly lower in streptomycin-treated mice than that in the control ones in Rev-2-3 (*p* = 0.04), Rev-2-4 (*p* = 0.041), Rev-2-5 (*p* = 0.002), and Rev-2-6 (*p* = 0.033) (session 23–27; two-way repeated measures ANOVA with Bonferroni correction; Figure 3B). In the third reversal stage (Rev-3), the first-choice never-rewarded rates of the first session (Rev-3-1; session 31) in control mice and streptomycin-treated mice were increased to 58.9 ± 3.6 and 52.5 ± 5.0%, respectively, compared to the final session of the Rev-2, and these rates dropped to below 30% from the seventh session (Rev-3-7; session 37) and the sixth session (Rev-3-6; session 36), respectively. In the fourth reversal stage (Rev-4), the first-choice never-rewarded rates of the first session (Rev-4-1; session 41) in control mice were increased to 51.4 ± 9.6% compared to the final session of the Rev-3 (Rev-3-10; session 40). In contrast, these rates in streptomycin-treated mice were only increased to 41.8 ± 6.6%, which were lower than chance level. These rates in control mice and streptomycin-treated mice dropped to below 30% from the seventh session (Rev-4-7; session 47) and the sixth session (Rev-4-6; session 46), respectively (Figure 3A and Supplementary Table 3).

By the intragroup comparison of the first-choice never-rewarded rates in the first to third sessions of each stage (Rev-1 to Rev-4), these rates of Rev-2 were significantly lower than the chance level (*p* = 0.001, Student’s one-sample *t*-test) and tended to be lower than that of Rev-1 and Rev-3 in the streptomycin-treated mice, although there was no difference in the control mice (Figure 3C).

Re-entry rates, defined as the number of incorrect first nose-pokes the same spot as the previous correct spot within the first 100 trials in each session, decreased as the trial progressed in both groups (Supplementary Figure 2). However, in a comparison of these rates in the first three sessions of each reversal stage (the averages of Rev-1-1 to 3, Rev-2-1 to 3, Rev-3-1 to 3, and Rev-4-1 to 3) (Figure 3D), re-entry rates in Rev-2 were higher in streptomycin-treated mice than in the control ones (*p* = 0.051, two-way repeated measures ANOVA with Bonferroni correction).

#### Comparison of the Response Speed to Reversal Tasks in the Reversal Stage

The first reversal stage (Rev-1) is the most important to judge cognitive flexibility, because all mice had no experience of reversal learning until Rev-1. Therefore, the influence of the alteration of the intestinal microbiome by streptomycin on cognitive flexibility was analyzed in detail using the data from Rev-1. Cumulative diagonal correct count and cumulative diagonal error count, defined as cumulative count of nose-poking to the two rewarded spots on the correct diagonal line (Figure 1F) within the first 150 choices in each session and the cumulative count of nose-poking to the two never-rewarded spots on the incorrect diagonal line within the first 150 choices in each session, respectively, are shown in Figure 4A. In the first session of reversal learning (Rev-1-1; session 11), the cumulative diagonal error count was higher than the cumulative diagonal correct count in both groups. In the second session of Rev-1 (Rev-1-2; session 12), cumulative diagonal error count became lower than cumulative diagonal correct count in control mice, although this count was continuously higher than cumulative diagonal correct count in streptomycin-treated mice. In the streptomycin-treated mice, reversal of these counts occurred at the third session of Rev-1 (Rev-1-3; session 13). In addition, to compare the speed of adaptation to the first reversal task, the never-rewarded rates of the first 40 trials that were completed by all mice were compiled for every five-trial block in the first session of Rev-1 (Rev-1-1) was analyzed. Error reduction significantly lower than chance level (50%) was observed in 21–25 (*p* = 0.022), 26–30 (*p* = 0.036), and 31–35 (*p* = 0.014) trials in the control mice (<40%; Student’s one-sample *t*-test). In contrast, significant higher level than chance level continued until 16–20 trials and this value did not decrease below chance level until 31–35 trails in streptomycin-treated mice (Figure 4B). These results show that the adaptation to the first reversal stage was faster in the control mice than in the streptomycin-treated mice.

#### Fecal Microbiota by 16S rRNA Gene Amplicon Sequencing Analysis

**FIGURE 5:**
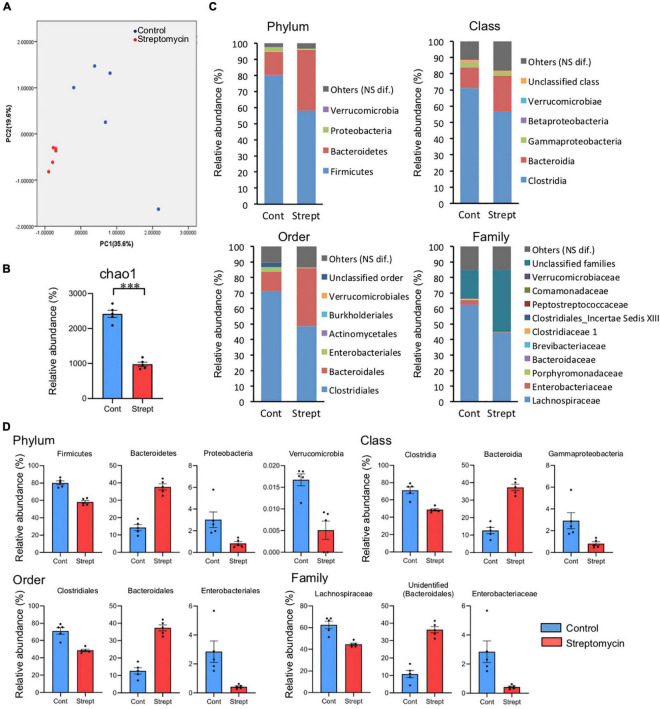
Comparison of relative abundance between control and streptomycin-treated mice. **(A)** Principle component analysis of the profiling data from fecal microbiota at the genus level. **(B)** Diversity analysis (Chao 1) of fecal microbiota. **(C)** The relative abundance of fecal microbiota at phylum, class, order, and family levels, expressed in percentage. Bacterial names showing significant difference between both groups are listed (*q* < 0.05, Mann–Whitney *U*-test corrected using the Benjamini–Hochberg method). **(D)** The comparison of relative abundance of predominant bacteria, which may be related to cognitive flexibility. The relative abundance of predominant bacteria in control and streptomycin-treated mice are shown in blue and red, respectively. All shown bacterial groups demonstrate significant difference.

In total, 342,707 reads (28,853–40,529 reads per sample) were obtained by 16S rRNA gene amplicon sequencing analysis and selection using the QIIME pipeline. A difference was observed in the fecal microbiota between control mice and streptomycin-treated mice. Streptomycin-treated mice shared similar microbiota (Figure 5A), and a reduction of microbial diversity was found in these mice (Figure 5B). The relative abundances of bacteria detected by 16S rRNA gene amplicon sequencing are shown in Supplementary Table 1. There were significant differences in 4 phyla, 6 classes, 9 orders, 16 families, and 26 genera between control and streptomycin-treated mice (*q* < 0.05, Mann–Whitney *U*-test corrected using the Benjamini–Hochberg method) (Figure 5C). Interesting differences of bacterial group were detected between these groups (Figure 5D). At the level of phylum, relative abundances of Firmicutes and Proteobacteria were lower, and that of Bacteroidetes was higher, in streptomycin-treated mice than in control mice. At the level of class, relative abundances of Clostridia and gamma-proteobacteria were lower, and that of Bacteroidia was higher, in streptomycin-treated mice than in control mice. At the level of order, relative abundances of Clostridiales and Enterobacteriales were lower, and that of Bacteroidales was higher, in streptomycin-treated mice than in control mice. At the level of family, relative abundances of Lachnospiraceae, Clostridiaceae 1, and Enterobacteriaceae were lower, and that of an unidentified family of Bacteroidales was higher, in streptomycin-treated mice than in control mice. At the level of genus, regarding predominant genera, only unidentified genera (an unidentified genus in Lachnospiraceae and an unidentified genus in unknown family of order Bacteroidales) showed significant differences, although many minor genera (under 0.5%) showed significant differences (Supplementary Table 1). All sequence data were deposited in the DDBJ Sequence Read Archive database under the accession number DRA009246 https://ddbj.nig.ac.jp/resource/sra-submission/DRA009246.

## Discussion

This experiment established an assessment task of cognitive flexibility using a touch screen operant system and evaluated the effect of intestinal microbiota on cognitive flexibility. In touch screen operant tests, the reward is the behavioral motive, and unlike pain avoidance behavior of Morris’s water maze test, there is high possibility for extrapolation to humans. Additionally, this test provides more data, not just simple correct/incorrect data, from its behavioral records (Horner et al., 2013). The task performed in this study was developed by mimicking the cognitive flexibility assessment task in IntelliCage (Endo et al., 2011) and, unlike the existing task, used a touch screen operant system as a visual discrimination task. In brief, the test of IntelliCage is composed of a “behavioral sequencing task,” which includes spatial learning with the acquisition of a behavioral sequence of shuttling between two corners, followed by “serial reversals.” This test can estimate two factors in cognitive flexibility. First, by finding a similar learning score with the original learning, we can confirm the mice performance and effect of treatment such as antibiotics. Second is the reversal learning-set, which indicates that animals can learn to learn (the formation of a learning-set). The concept of learning-set refers to the ability of progressive shift from trial-and-error to the immediate solving of new problems on the basis of previously learned experiences, and is thought to be an executive function of the brain. The test paradigm was originally developed on the basis of the idea of the Brixton spatial anticipation task, which has been utilized as a clinical assessment method for human executive function (Bielak et al., 2006; Wood and Liossi, 2007).

The data obtained from this task were compared with data from the cognitive flexibility assessment task by IntelliCage (Endo et al., 2011). A decrease in first-choice never-rewarded rate at the acquisition stage and the daily decrease in this rate within each stage (intra-stage) and the trial number-dependent decrease in the first-choice never-rewarded rate within each session (intra-session) at the serial reversal stage were similar to those of the cognitive flexibility assessment task in IntelliCage, demonstrating that a touch screen operant system task was successfully constructed to evaluate mouse cognitive flexibility. IntelliCage is a system in which mice learn the corners where rewards can be obtained by their instinctive searching behavior for water in a three-dimensional space in their normal living environment, and the correct place is the same as the place where mice get rewards. In the touch screen operant system, the mice learn the position to obtain food on a two-dimensional touch screen, which deviates from instinctive food-seeking behavior in an inexperienced environment (chamber) that is different from their normal living environment. Additionally, the right spot to touch dose not provide the reward; the reward is given by a dispenser after exhibiting the correct behavior. Therefore, although these are test systems that similarly utilize appetite as a positive reinforcer, the touch screen operant system has more non-routine and non-instinctive elements for mice than the IntelliCage test, making it difficult to establish a behavioral sequence. Nevertheless, it is significant that the assessment task of cognitive flexibility using a touch screen operant system obtains results similar to those of the behavioral sequencing task and serial reversals used in the IntelliCage system. However, the present study revealed differences in the values of first-choice never-rewarded rate (approximately 20%) in the acquisition stage, which were higher than those in which IntelliCage decreased to nearly 5% (Endo et al., 2011), and the adaptation to the serial reversal task that was observed in IntelliCage was not observed in this study (the phenomenon in which the first-choice never-rewarded rate of the first session in each stage decreased gradually due to the repetition of reversal). This may be due to the difference between the touch screen operant system and the IntelliCage system, but further investigation is needed to increase the number of serial reversal tasks.

In rodents, a test using a touch screen operant system has been reported to be effective in assessing cognitive impairment in human disease model animals, including those for AD, schizophrenia, aging, and exposure to stress (Brigman et al., 2008, 2009; Karlsson et al., 2009; Romberg et al., 2013; Yang et al., 2015). Rodent cognitive tests using touch screen operant systems include visual discrimination, object-location paired association, and location discrimination of pattern separation tasks (Horner et al., 2013; Oomen et al., 2013). In visual discrimination tasks, a combination of visual discrimination and reversal learning is used (Brigman et al., 2008, 2009; Karlsson et al., 2009; Romberg et al., 2013; Yang et al., 2015). The mouse discriminates and learns the correct and incorrect visuals, and behavior adaptability is evaluated when the correct and incorrect visuals are reversed. The object-location paired association is a form of learning that is associated with two different stimuli and tasks assess learning scores by a combination of a visual stimulus and its location on the touch screen (Horner et al., 2013). In the location discrimination task of the pattern separation tests, the mouse recognizes the distance between two or more target windows and learns their correct positions (Oomen et al., 2013). This location discrimination is reported to primarily use as a functional evaluation system of the hippocampal dentate gyrus, which is involved in spatial memory (Gilbert et al., 2001; Leutgeb et al., 2007; Bakker et al., 2008; Clelland et al., 2009). Among these test protocols, tests using a combination of visual discrimination tasks and reversal learning are used. However, these test protocols have been derived from simple discrimination, and it is only a task to learn new correct visuals after a change. It can be thought to be not sufficient to assess cognitive flexibility, which is the ability to recognize changes in existing rules and to formulate and apply new rules. In the present study, mice showed a progressive improvement in serial reversals, possibly because of behavioral sequencing-based reversal tasks as well as the previous study in IntelliCage (Endo et al., 2011, 2012).

Next, we investigated the effects of intestinal microbiota on cognitive flexibility from data comparisons between the control and streptomycin-treated mice. Although both groups showed the acquisition of a behavioral sequence of shuttling between two corners and the adaptation to serial reversal learning, the streptomycin-treated mice were delayed in learning the reversal of cumulative diagonal correct count and cumulative diagonal error count in the Rev-1 (Figure 4A) and the timing of the decrease in the never-rewarded rate of Rev-1-1 (Figure 4B), indicating that dysbiosis by streptomycin induced a decline in cognitive flexibility. It is reported that aminoglycoside drugs including streptomycin are poorly absorbed in the healthy gastrointestinal tract (Germovsek et al., 2017). Therefore, the possibility of orally administered streptomycin directly affecting the brain is low; however, the transfer of streptomycin from the intestinal lumen to the central nervous system should be analyzed using highly sensitive methods in the near future. To our knowledge, this is the first study to show that antibiotic-derived dysbiosis causes cognitive decline, including a decrease in cognitive flexibility, in spatial learning tests. The study by Frohlich et al. (2016), which is the only similar study, reported no effects in a spatial learning test with the Barnes maze in mice treated with antibiotics. We believe that this difference between Frohlich’s result and ours can be attributed to the sensitivity of our touch screen task than that of the traditional Barnes maze task.

In the streptomycin-treated mice, the first-choice never-rewarded rate of Rev-2 was lower than that of Rev-3 (Figure 3C), showing a difference from the normal learning curve that gradually changes by the repetition of reversal learning as a result of control mice. Furthermore, this rate of only Rev-2 in streptomycin-treated mice was lower than that in the control ones (Figure 3B), indicating that this phenomenon is caused by the “perseveration” to the correct diagonal line learned in the acquisition stage (correct diagonal line of Rev-2 was same as that of acquisition stage). Additionally, streptomycin-treated mice showed a higher re-entry rate at the acquisition stage and the reversal stage compared with the control mice (Figures 2B,C, 4D), confirming that streptomycin-treated mice more frequently repeated nose-pokes to the previous correct position. This repeat behavior seems to be relevant to the “persistence” in human dementia, suggesting that dysbiosis induces persistence. This is the first report to show that intestinal microbiota affects perseveration or persistence. Thus, we could evaluate the difference in perseverance and persistence, which are signs of a decline in cognitive flexibility, by detecting the subtle quantitative and qualitative differences in mice behavior as experimental data, indicating that touch screen operant system task developed in this study is extremely sensitive and can be applied to the detection of slight changes or differences in cognitive flexibility. This task is very useful in the field of cognitive function and dementia, since beginning treatment at the early stages of decline of cognitive flexibility is important for the prevention of dementia.

Very few studies have reported the relationship between the intestinal microbiome and cognitive flexibility. Liu et al. (2019) compared the intestinal microbiome of three groups of AD patients, amnestic mild cognitive impairment (aMCI) patients, and healthy subjects in a Chinese cohort, and reported that the relative abundance of Firmicutes in AD patients was lower than that in healthy subjects, and Bacteroides was higher in aMCI patients than in healthy subjects (there was no significant difference between healthy subjects and AD patients). These data show the same trend in performance-dependent cognitive flexibility as the results of the present study, in which the relative abundances of Firmicutes and Bacteroides were lower and higher, respectively, in the streptomycin-treated mice with less cognitive flexibility. Similar cognitive flexibility-dependent differences in the intergroups were observed in both studies as follows: Clostridia (class), Clostridiales (order), Lachnospiraceae (family) in the Firmicutes and Bacteroidia (class), Bacteroidales (order), Bacteroidaceae (family) in the Bacteroidetes. These results strongly suggest that bacteria belonging to these two major phyla are involved in cognitive flexibility. However, this study also produced data of a minor bacterial group that was not consistent with our results: Proteobacteria (phylum), Gammaproteobacteria (class), Enterobacteriales (order), which were lower in the streptomycin group in our experiment, but were significantly higher in AD patients. Using the reversal task of the Morris water maze, Magnusson et al. (2015) reported that, in mice with intestinal microbiota altered by the ingestion of a high-fat or high-sucrose diet, Clostridiales (order) and Bacteroidales (order) were negatively and positively correlated, respectively, with cognitive flexibility. The relationship between these bacterial groups and cognitive flexibility contradicts this finding. We posit that the mice of Magnusson et al.’s (2015) study may have been influenced by factors other than intestinal microbiota; that is, overingesting of lipids and sucrose, and the obesity induced by these diets. In fact, epidemiologic studies of more than 8,500 pairs of twins have shown that obesity increases the risk of dementia (Xu et al., 2011). We consider that our data is more accurate for examining the relationship between cognitive flexibility and intestinal microbiota than that of Magnusson et al. (2015), because there was no difference in body weight between the groups during the study period in our study and it is unlikely that physiological relevant concentrations of streptomycin reached the brain due to poor absorbance from the gastrointestinal tract.

In this study, we established a cognitive flexibility assessment task consisting of a behavioral sequencing task with spatial learning and serial reversal learning in touch screen operant system. Using this task, we found that the composition of intestinal microbiota influences cognitive flexibility. This task will be meaningful for future studies concerning cognitive flexibility in mice, because it allows real-time imaging of brain activity during task performance with a micro-endoscope, and surgical intervention during the test period, which is not possible with the high-throughput, automated system, IntelliCage. A limitation of the present study is that mice demonstrated only slight adaptation to the serial reversal task (the phenomenon in which the first-choice never-rewarded rate of the first session in each stage decreases gradually due to the repetition of reversal). Further studies are required to improve the test protocol in order to advance our understandings of relationships between cognitive flexibility and relationships between brain cognition and gut microbiota.

## Data Availability Statement

The datasets presented in this study can be found in online repositories. The names of the repository/repositories and accession number(s) can be found in the article/Supplementary Material.

## Ethics Statement

The animal study was reviewed and approved by the Kyodo Milk Industry Animal Use Committee.

## Author Contributions

HT, MK, and MM designed the study. HT performed the experiments. YM supported the experiments. HT, KI, MK, and MM interpreted the data. HT and KI statistically analyzed the data and wrote the Materials and Methods and Results. KI wrote the Introduction and Discussion. KI and MM drew the figures. DJ and TS revised the manuscript. MK and MM critically revised the manuscript. All authors contributed to the article and approved the submitted version.

## Conflict of Interest

HT, KI, and MM were employed by Kyodo Milk Industry Co., Ltd. The remaining authors declare that the research was conducted in the absence of any commercial or financial relationships that could be construed as a potential conflict of interest.

## Publisher’s Note

All claims expressed in this article are solely those of the authors and do not necessarily represent those of their affiliated organizations, or those of the publisher, the editors and the reviewers. Any product that may be evaluated in this article, or claim that may be made by its manufacturer, is not guaranteed or endorsed by the publisher.
