# Supplementary material for: Impact of Intestinal Microbiota on Cognitive Flexibility by a Novel Touch Screen Operant System Task in Mice
Source: Front Neurosci. 2022 Jun 23;16:882339. doi: 10.3389/fnins.2022.882339 (PMC9259885; doi:10.3389/fnins.2022.882339)
Supplement: Supplementary file 1 [file Data_Sheet_1.pdf]

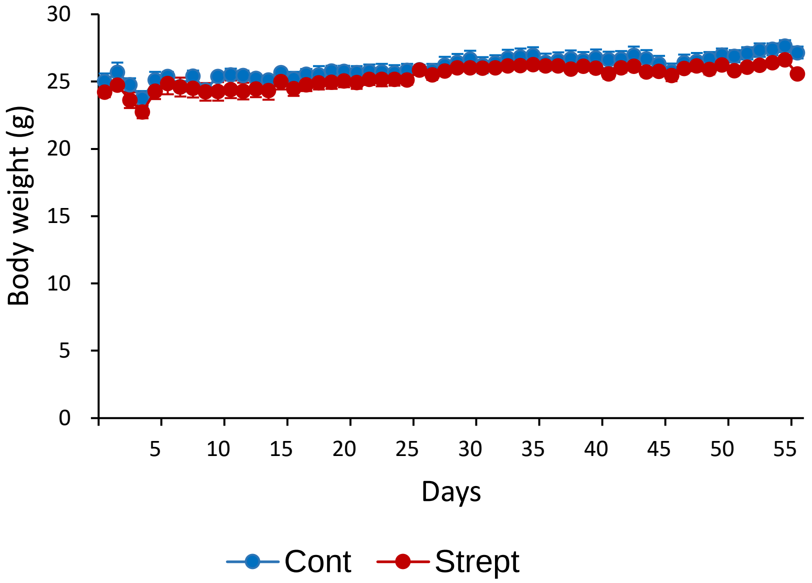

Supplementary Figure S1. Change in body weight

A significant difference was not observed in body weight between the two groups. Temporary weight loss, which was less than 80% of body weight of non-tested mice, was observed in both groups at the start of the task (days 3 and 4) but was recovered within a few days. Error bars represent SEM.

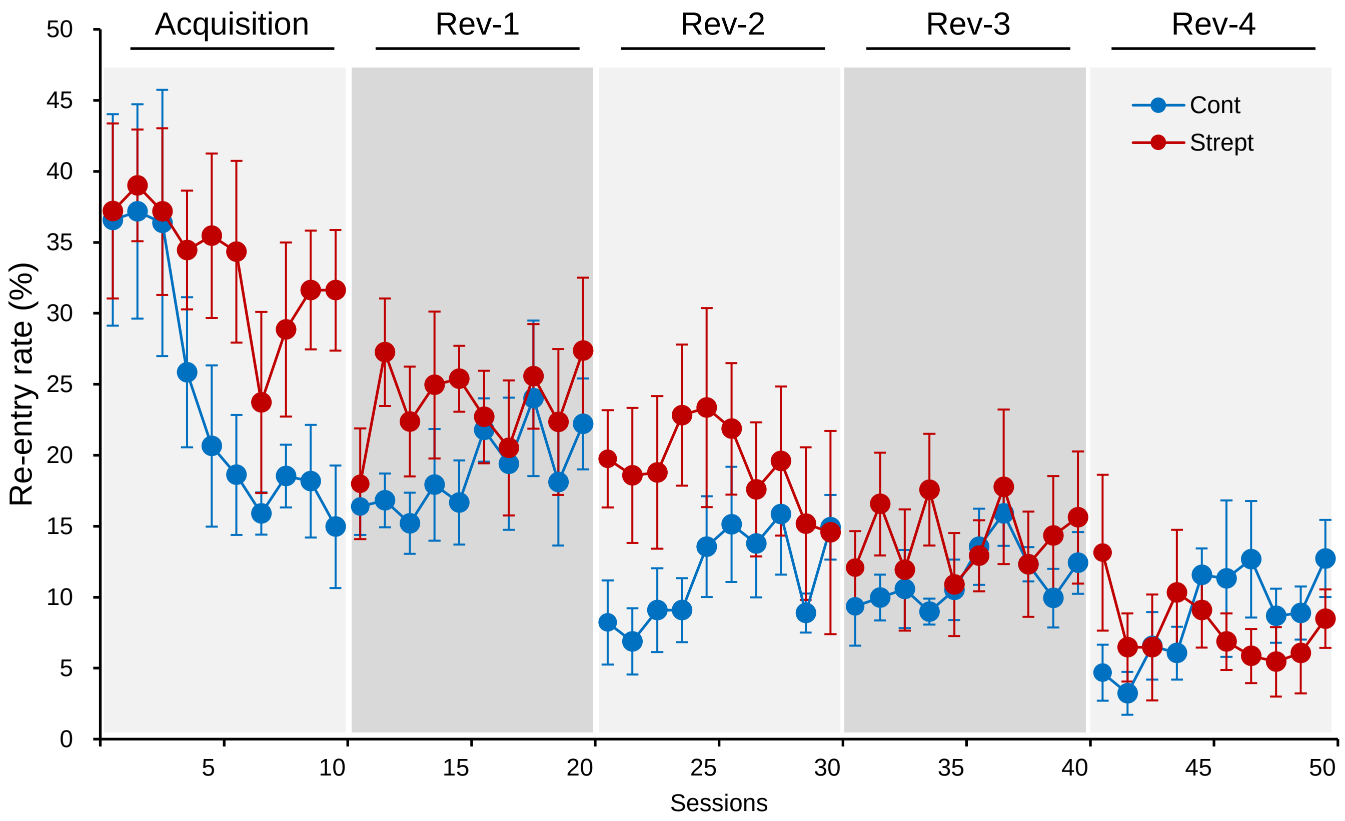

Supplementary Figure S2. Comparison of the Re-entry rates
Error bars represent SEM. Comparison data of this rate in the final three sessions of acquisition phase (the averages of Acq-1-8 to 10) and the first three sessions of each reversal stage (the averages of Rev-1-1 to 3, Rev-2-1 to 3, Rev-3-1 to 3, and Rev-4-1 to 3) are shown in Figure 2c and Figure 3d, respectively.
